# Nature-human’s celebratory ties: indigenous foodways and festive traditions of central India

**DOI:** 10.1186/s13002-026-00904-6

**Published:** 2026-05-22

**Authors:** Akash Kumar Srivastava, Akanksha Yadav, Vinita Chandra

**Affiliations:** 1 Department of Humanistic Studies, Indian Institute of Technology (BHU) Varanasi, Varanasi, India; 2 Amity School of Business, Amity University, Jharkhand, India

**Keywords:** Central India, Indigenous identity, Nature-based festivals, Ritual foodways, Symbiosis with ecology

## Abstract

The region of central India is home to several indigenous groups, living in forested areas, who show their close affinity to their land through various cultural practices. We found that almost every activity they perform revolves around their connection to nature, exhibiting their reverence for Mother Earth and other natural entities with which their lives are interrelated. Food-centred festivals are notable among these pursuits, as they provide occasions to commemorate ancestral customs, celebrate agricultural cycles, and reinforce the community’s devotion to nature. It may be observed that food plays a key role in shaping cultural settings. The present study aims to document these events and highlight the symbolic connotations of organising such festivals. Derived from the immersive fieldwork in the villages of Maikal Hills, the festivals, which frequently fall on significant agricultural events, show how the festive events are an important constituent of the region’s foodways. This study examines the embedded aspects of these foodways through the lens of environmentalism and indigenous identity. We have found that such celebrations are based on a holistic worldview in which food embodies the community’s symbolic reverence for nature. At the same time, it is the primary source of nutrition and shapes cultural identity.

## Introduction

Food practices provide an important lens through which relationships between human societies and their environments can be understood. In many indigenous communities, food is not only a source of subsistence but also a medium through which cultural identity, ecological knowledge, and ritual beliefs are expressed. Festivals centred on food play a particularly significant role in this process because they bring together ritual, agriculture, and community participation. Through these events, food becomes more than nourishment; it acquires symbolic meanings that reflect social values, environmental ethics, and collective memory. With this backdrop, in the present ethnographic work, we explore the celebratory aspect of foodways, shedding light on how festivals attribute meanings and on how foods not only feed humans but also shape their cultural settings, identities, and moments of leisure. Scholars have noted that these practices express a deep sense of environmental interdependence and reinforce communal ties to land and resources [16]. Food-centred festivals therefore function as cultural spaces where ecological knowledge is transmitted across generations and where communities reaffirm their connections with the natural world.

In central India^1^, groups like Baiga, Gond, Bhil, and others maintain strong relationships with their surrounding landscapes, which include forests, rivers, and mountains. These landscapes are not perceived merely as physical environments but as culturally meaningful spaces inhabited by ancestral spirits and protective deities. Ritual practices, myths, and oral traditions of these tribal groups^2^ frequently reflect this close relationship with nature [2, 5, 7, 24]. Agricultural activities, forest gathering, and seasonal celebrations are therefore embedded within broader systems of ecological knowledge and spiritual belief.

Here, the festivals that largely deal with foodways^3^ are particularly important as they all include social connectedness, ancestral honouring and environmental sustainability. These celebrations typically involve ritual offerings of locally cultivated grains, forest produce, and seasonal foods. They mark important agricultural transitions while simultaneously reinforcing community solidarity and ancestral remembrance [3, 10, 23, 26]. Through these practices, food becomes a symbolic medium through which relationships between humans, nature, and the spiritual realm are expressed.

Considering this, the present study examines such festivals to understand how food is a part of humans’ everyday cultural practices and in what ways the symbolic meanings of these events reinforce the ritual and ecological ties of central Indian tribal communities. Through the use of ethnographic methods, involving field immersion^4^ in the Maikal Hills region, we show how the celebrations serve as expressions of Indigenous environmentalism and cultural heritage, as well as draw attention to their relevance for people in a context of modernity and environmental change [29].

This study also engages with the material culture of these events and considers tools, spaces, and ingredients to divulge their subtle meanings. It ponder on that clay pots, bamboo baskets, and stone grinders are not just functional material objects but also cultural moments of continuity, as Miller (2005) [27] and Gell [18] emphasised. Similarly, the spaces where these festivals occur, like village open spaces, a courtyard in a house, a sacred place on a mountain, or riverbanks, all serve as stages for communal performances that underpin tribal identity and fortify the bond with nature [36, 37]. The ingredients used in these events, such as grains harvested from local fields, wild herbs gathered from nearby forests, seasonal fruits and hand-pounded spices, further reflect the intimate connections between indigenous food systems and surrounding ecosystems. By documenting these accounts from the fieldwork, this paper provides a comprehensive picture of food-centred festivals of central India (especially related to the Gond and Baiga communities inhabiting the Maikal Hills region) and presents them as multidimensional cultural practices by analysing these tangible elements alongside intangible aspects like songs and stories.

**Fig. 1:**
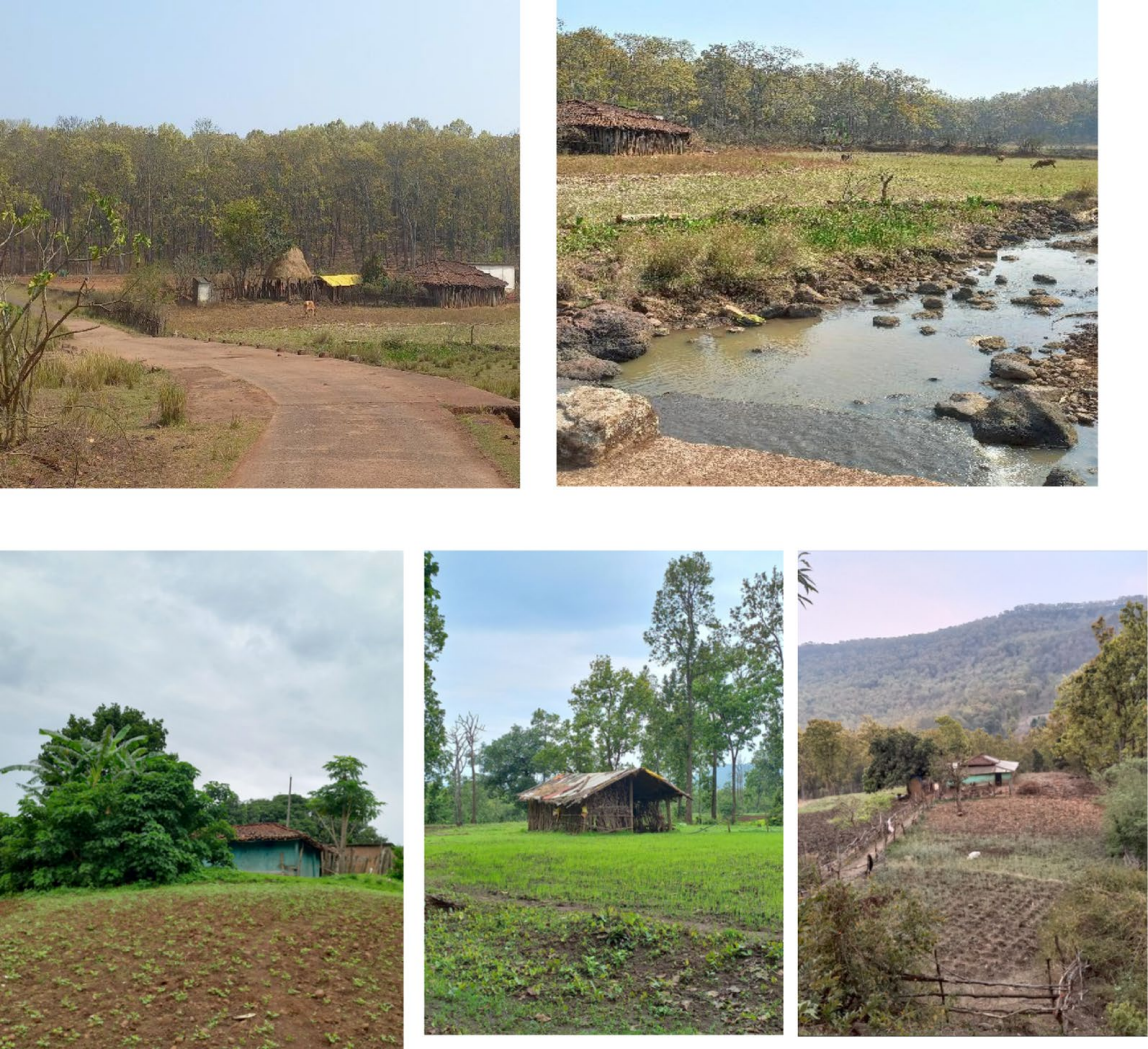
Tribal settlement amidst the forested landscape of Maikal Hills, Central India. (Source: First author)

It further sheds light on why the food-centred festivals of tribes in central India are the rich expressions of the intersection between ecology, culture, and society [19, 32]. The studied groups live in the remote forested landscape of the Maikal Hills (Fig. 1). They are considered to be the descendants^5^ of the early inhabitants of the region [6, 33] and many aspects of their material culture show similarity with the archaeological evidence from the region [15, 17, 31]. This scenario emphasises the continuity of past traditions in their living customs. Understanding the ritual role of food in these festivals, therefore, provides insight into how indigenous communities maintain ecological relationships and cultural continuity in changing environmental contexts. To situate these findings within a broader context of indigenous food traditions, the study also compares the festivals of the Maikal Hills with similar harvest and food rituals observed in other regions of India and in indigenous societies globally.

The study is guided by the following research objectives: (1) to document major food-related festivals and their associated ritual practices; (2) to analyse the symbolic and ecological meanings of ritual foods such as millets, mahua, forest produce, and newly harvested grains; and (3) to examine how these festivals reinforce indigenous environmental ethics and cultural identity in the context of changing ecological and socio-economic conditions. It is hypothesised that food-centred festivals function as important cultural mechanisms through which indigenous communities maintain reciprocal relationships with their environment while simultaneously strengthening food sovereignty and collective memory.

## Materials and methods used in the study

**Fig. 2:**
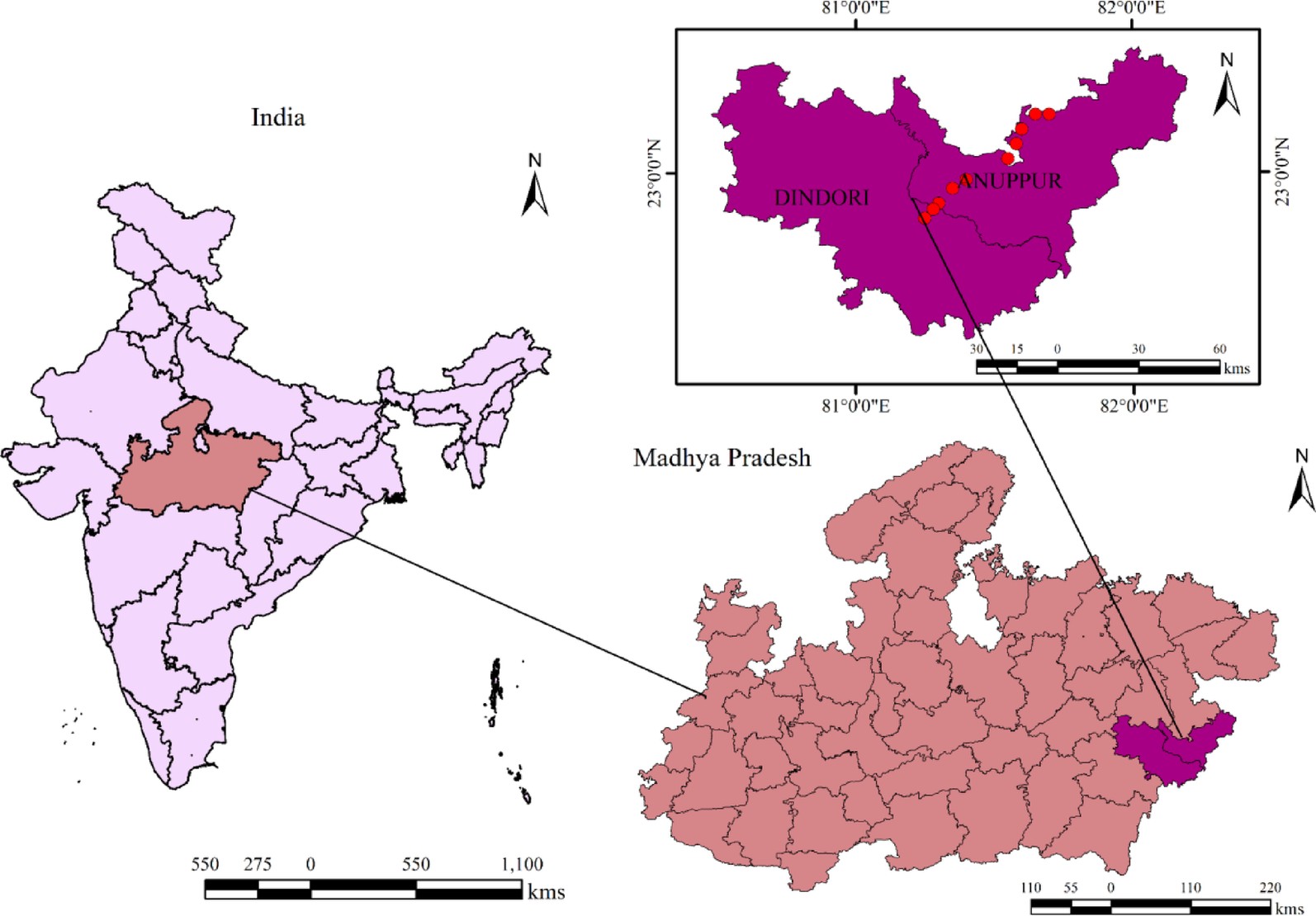
Map of the Maikal Hills region indicating the settlements visited during ethnographic fieldwork. (see Table 1 for details of locations and participants).

The study is based on ethnographic fieldwork conducted over a year, comprising three rounds of visits from November 2023 to December 2024, in villages across the Maikal Hills^6^, located in the Anuppur and Dindori districts of Madhya Pradesh. The geological and ecological features of the Maikal hills are marked by a rich flora and fauna, several rocky plateaus, numerous streams, and deep gorges. This territory, which is the meeting place of the Vindhyan and Satpura mountains, is important for a number of reasons, one of which is the high concentration of Indigenous peoples who have lived in central India for ages (Fig. 2).

The Gonds and Baigas, the two most prominent indigenous populations on the subcontinent, are mostly concentrated here [4, 14, 22, 35], whose lives and worldviews are largely centred on forests, mountains, and rivers. In this regard, the primary contact groups for the ethnographic information were the Gond and Baiga, who are nestled in this densely forested place and are distanced from non-tribal people, where they practice their traditional customs and rituals, along with an ancient manner of subsistence. The ethnographic fieldwork was conducted across several Gond and Baiga settlements in the Maikal Hills region. The geographical and socio-demographic characteristics of the study sites and participants are summarised in Table 1.

**Table 1:** Fieldwork locations in the Maikal Hills region of central India and demographic characteristics of study participants involved in the ethnographic research

Settlement	GPS coordinates (Approx.)	Altitude (m)	Ecology/Landscape type	Ethnicity (Self-identified)	Language (Glottolog classification)	Religion (Self-declared)	No. of inhabitants (Approx.)	Study participants (Gender, age, Socio-demographic profile)
Farri Semar	23.23° N, 81.70° E	~ 540	Mixed deciduous forest with upland agriculture	Gond	Gondi (Dravidian; Glottolog: gond1247)	Indigenous beliefs with Hindu influence	~ 850	7 participants (4 M, 3 F; age 32–70; farmers, ritual participants)
Damgarh	23.20° N, 81.65° E	~ 520	Forest plateau with seasonal streams	Baiga	Baiga (Indo-Aryan; Glottolog: baig1242)	Indigenous animistic traditions	~ 620	6 participants (3 M, 3 F; age 28–68; cultivators, forest gatherers)
Mekal Pahar	23.15° N, 81.60° E	~ 600	Hilly forest terrain with shifting cultivation	Gond & Baiga	Gondi/Baiga	Indigenous traditions	~ 700	5 participants (3 M, 2 F; age 35–72; elders, farmers)
Mirchadadar	23.10° N, 81.58° E	~ 550	Sal-dominated forest and agricultural clearings	Baiga	Baiga	Indigenous beliefs	~ 480	6 participants (3 M, 3 F; age 30–65; cultivators, ritual specialists)
Choktipani	23.05° N, 81.55° E	~ 530	Forest-agriculture mosaic landscape	Gond	Gondi	Indigenous beliefs	~ 560	5 participants (3 M, 2 F; age 29–64; agricultural households)
Sonteerath	22.98° N, 81.40° E	~ 510	Riverine landscape with forest patches	Baiga	Baiga	Indigenous traditions	~ 450	6 participants (3 M, 3 F; age 27–67; farmers, community elders)
Kharideeh	22.95° N, 81.35° E	~ 520	Mixed forest and cultivated uplands	Gond	Gondi	Indigenous beliefs with Hindu influence	~ 620	7 participants (4 M, 3 F; age 33–71; farmers, ritual leaders)
Bajag	22.90° N, 81.30° E	~ 505	Agricultural plains near forest fringe	Gond	Gondi/Hindi dialect	Hindu and Indigenous practices	~ 1100	8 participants (5 M, 3 F; age 25–60; cultivators, traders)
Jagatpur	22.88° N, 81.28° E	~ 500	Forest-edge settlement	Baiga	Baiga	Indigenous traditions	~ 420	7 participants (4 M, 3 F; age 31–69; farmers, forest product collectors)
Barbaspur	22.85° N, 81.25° E	~ 495	Mixed deciduous forest with scattered farms	Gond	Gondi	Indigenous beliefs	~ 500	8 participants (4 M, 4 F; age 30–68; cultivators, household food preparers)

Coordinates and altitude values are approximate and provided to indicate the geographical context of the study area

**Fig. 3:**
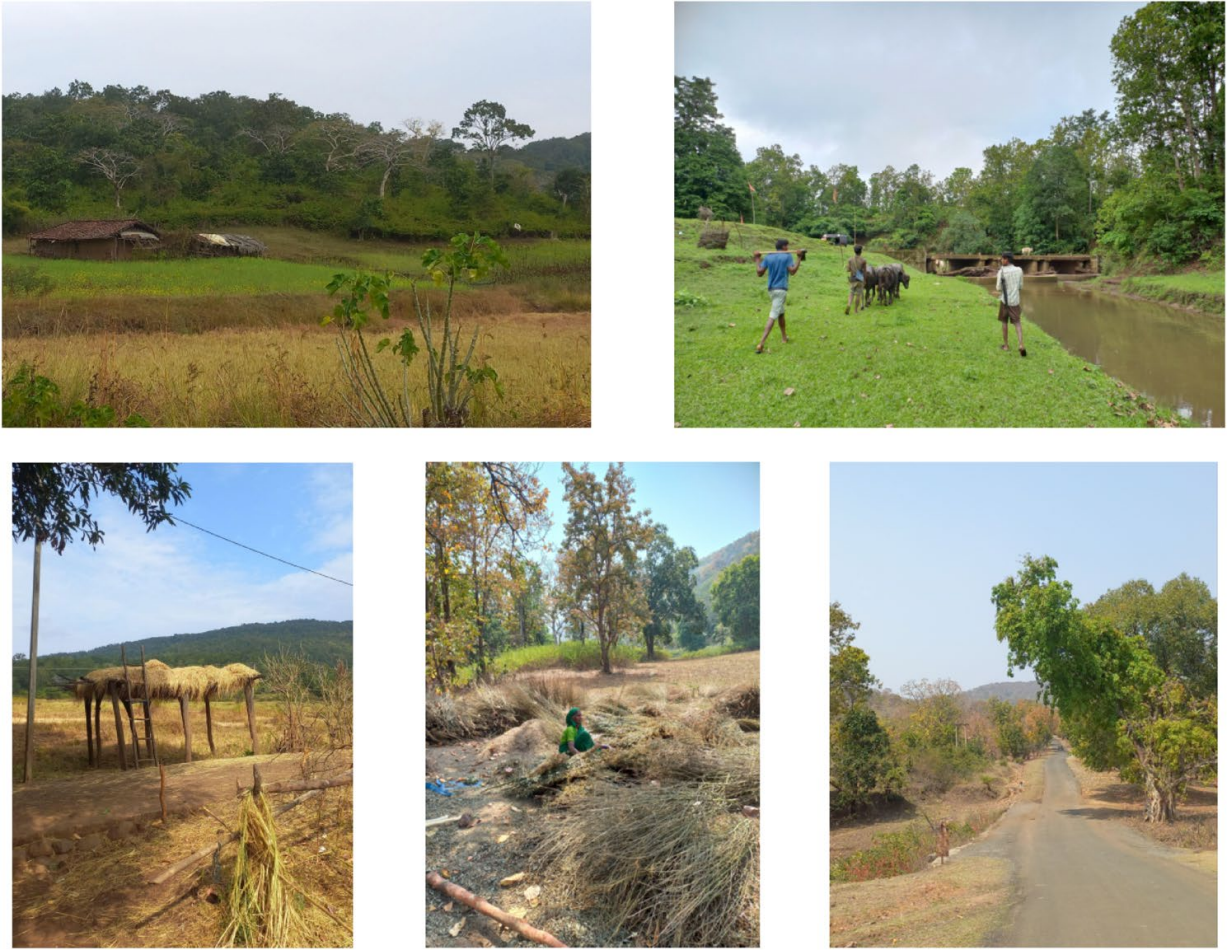
Tribal lifeways around the Mountainous landscape of Maikal Hills, Central India (Source: First author)

Data collection for this study involved semi-structured interviews, non-participant observation, and informal conversations with community elders, ritual specialists, and women involved in food preparation. All interviews were conducted in the local dialect of these communities, i.e. *Chhattisgarhi*, with the aid of an interpreter. This involved 40 household surveys and semi-structured interviews with 65 individuals from both groups. Here, we used the snowball sampling method^7^ to choose the respondents, where the inclusion criteria were their status as forest dwellers and high adherence to the traditional way of life (Fig. 3). In this fieldwork, the whole aspect of their foodways was documented to understand the Indigenous way of fulfilling the subsistence demands, where the ethnic identity is reflected in the patterns of the Indigenous food system. Everything was recorded through open-ended questions and participation in the day-to-day activities of these people. Hence, non-participant observation, or, in some sense, everyday immersion, became the cornerstone of this study, providing a more objective view than participant observation.

Within this series of information, the festivals and customs associated with different foodways-related occasions possessed a compelling uniqueness and drew us in effortlessly. Amidst all the information shared about the foodways activities in the indigenous setting, this dataset emerged as the most intriguing and attention-grabbing. After that, we pursued this objective further and covered most of these festivals. Some of the ritual events were documented using field notes, audio recordings, and photographs, with the respondents’ consent. While there are many such events, for the purpose of this paper, we have included only food-related festivals of the Gond and Baiga communities. These festivals are celebrated in different seasons of the year. We personally documented a few events that occurred during the fieldwork months. However, we also included all such festivals related to the objective of this paper through telephonic interviews with locals during those festivals, leveraging our existing connections.

As the region of Maikal Hills is snuggled in the forested landscape and is inundated by rivers like the Narmada, Son and their several tributaries, which originate from here. These communities, who are living in this bountiful realm, have developed a great relationship with their surroundings. And when some scholars say they are the descendants of the region’s early dwellers [6, 33], it is undeniable that each aspect of their everyday life is the result of long-term interaction with the same ecosystem. Therefore, moving ahead with the methods of analysis, we contextualised our work within the existing scholarly framework. In conjunction with the collected ethnographic data, we consulted the existing literature on the Indigenous groups of central India, drawing on some anthropological theories discussed later in this paper. After combining all the information, these festivals are further synthesised in depth, so that the clues come to the fore on how the associated food system leverages human-nature ties.

For the comparative analysis of ritual food practices, the festivals documented were categorised according to the dominant type of ritual food used in each event, such as millets and local grains, forest-derived foods, or newly harvested crops. A simple frequency distribution was calculated based on the number of festivals in each category (*n* = 7). This basic quantitative comparison was used to identify patterns in ritual food use and to facilitate comparison with findings reported in previous studies on indigenous food festivals in other regions.

### Theoretical framework

Environmentalism, particularly in its anthropological form, challenges earlier views that separated human society from the natural world or framed the environment merely as a backdrop to cultural life. Tim Ingold [21] argued that humans do not stand *apart* from nature but exist *within* an ecological continuum, engaging in what he calls ‘dwelling’^8^ in the world. This perspective contests earlier structural or utilitarian interpretations that treated the environment as a passive resource to be exploited. Instead, Ingold’s view on environmentalism foregrounds the intimate, lived, and reciprocal relationships through which communities understand, inhabit, and sustain their landscapes. It says the environment is not an external domain, but a co-creating presence that shapes social life, beliefs, subsistence, and ritual practices. As our fieldwork reveals, acts such as offering the first millets to spirits, preparing seasonal tubers, or brewing mahua serve as ecological rituals that acknowledge interdependence with the forest and land. Rather than passive cultural traditions, these festivals are active engagements with landscape regeneration, biodiversity conservation, and sustainable subsistence.

Building from this, sensory anthropology expands the analytical frame by arguing that humans do not encounter the environment abstractly but through the corporeal immediacy of smell, sound, taste, touch, and sight [8, 20]. Sensory experience, far from being biologically uniform, is culturally patterned and historically grounded. Sensory theorists contend that landscapes are not simply ‘used’ but felt, perceived, and inhabited, creating what Sarah Pink [28] describes as an ‘*embodied emplacement*,’ a condition where memory, skill, and emotion converge in everyday practices. Sensory anthropology thus reveals how ecological intimacy is experienced through the body: the heat of the earth during cultivation, the scent of forest herbs, the tactile knowledge of soils, or the rhythmic sounds of rivers guiding foraging and fishing decisions. These sensory engagements generate a lived environmental knowledge that is both practical and deeply affective. Therefore, drawing on Sutton [38] and Howes [20], the present study interprets Adivasi^9^ festivals as sensorially saturated practices through which knowledge of crops, seasons, forest produce, and culinary traditions is remembered, transmitted, and emotionally reinforced, with a side-by-side connection to the ecology strengthened. Sensory experiences thus function as anchors of identity, linking the body, community, and environment.

Bringing these two perspectives together allows this study to conceptualise indigenous environmental relationships not as mere survival strategies but as holistic, embodied life-worlds. In this view, the idea of environmentalism highlights the structural and relational foundations of these life-worlds. It draws attention to how people understand themselves with the forest, through water, and within seasonal cycles. At the same time, sensory anthropology illuminates the intimate modes through which these relations are felt, reproduced, and communicated. Through sound, smell, touch, movement, and everyday practices, these relationships become lived and experienced realities. Together, these perspectives reveal environmental relations as deeply embedded in cultural, sensory, and social worlds rather than as simple strategies of resource use. They also reveal a continuum where ecological interdependence is continuously reinforced through ritual acts, culinary practices, craft traditions, and everyday subsistence. By using these perspectives, the study aims to reveal how Adivasi festivals embody a holistic worldview, offering insights into cultural resilience and ecological sustainability. Through the above framework, the study also demonstrates that these food festivals are not merely ritual celebrations but complex ecological performances through which cultural identity, environmental ethics, and sustainable relationships with the landscape are continually reproduced.

### Ethnographic overview of prominent food-centric tribal festivals

Throughout the ethnographic fieldwork in the Maikal Hills, we came across a diverse array of festivals centred around food that regulate the local tribal group’s agricultural and ceremonial lives. In conversations with several village elders, we documented oral traditions and ceremonial practices related to seven such events, reflecting a deeply embedded cultural ecology in which food, land, and ritual life are intricately linked. The principal food-centred festivals documented during ethnographic fieldwork, along with their ritual characteristics and cultural meanings, are summarised in Table 2.

**Table 2:** Main characteristics, ritual practices, and cultural meanings of food-centred festivals among Gond and Baiga communities

Festival	Season/Month	Main ritual foods	Key ritual practices	Description of the event	Cultural and ecological meaning
Aakha Teej (Akati)	April–May	Paddy grains	Offering paddy in palash leaf bowls at the shrine of Thakur Dev	Households carry bowls made of palash leaves filled with paddy to the village deity shrine where seeds are blessed before the sowing season	Symbolises the beginning of the agricultural year and expresses gratitude to the earth and protective deities for crop fertility
Bidari	May–July (pre-sowing season)	Millets (kodo, kutki), maize, pulses	Ritual mixing of seeds, offerings to deities, sacrificial rituals	Seeds of multiple crops are assembled and ritually consecrated by village priests before being distributed to households for sowing	Reinforces collective agricultural responsibility and invokes divine protection for crops and ecological balance
Hareli/Hariyali	July–August (monsoon)	Babara sweet dish; forest plants	Collection of ritual forest items and worship of agricultural tools	Household heads collect symbolic forest plants and perform rituals in fields and homes to honour the earth and farming tools	Celebrates agricultural fertility, honours livestock and farming implements, and recognises the role of forests in sustaining livelihoods
Bhojali	Monsoon (Savan Purnima)	Sprouted wheat or barley	Germination of grains in earthen pots and immersion in water bodies	Young girls nurture sprouting grains for several days and later immerse them in rivers or ponds during a community procession	Symbolises fertility, regeneration, and the cyclical renewal of life and crops
Chherata	Winter (December–January)	Rice, pulses, parched grains	Children collect grains from households and cook communal food	Children visit homes singing ritual chants and collecting grains, which are later cooked and shared collectively after ritual offerings	Reinforces community solidarity and reflects agricultural myths associated with the origins of cultivation
Nawakhani	Harvest season (Bhado month)	Newly harvested grains	Offering the first harvest to ancestors and deities	A small portion of newly harvested crops is cooked and ritually offered before families consume the new produce	Expresses gratitude for agricultural bounty and acknowledges ancestral guardianship over crops and land
Javanra	Spring ritual cycle	Barley and wheat sprouts	Installation of sacred soil and germinating seeds with Jyoti Kalash	Seeds are sown in ritual soil baskets and worshipped for nine days before immersion in nearby water bodies	Represents agricultural renewal, divine blessing, and the sacred relationship between land, fertility, and community

The festivals summarised in Table 2 demonstrate how ritual food practices are closely embedded within ecological cycles and agricultural rhythms in the Maikal Hills region. From the perspective of environmental anthropology, these events illustrate the reciprocal relationship between indigenous communities and their surrounding landscapes, where food offerings and seasonal rituals acknowledge the interdependence of humans, crops, forests, and spiritual entities. At the same time, these celebrations involve sensory engagements, such as the taste of newly harvested grains, the aroma of fermented mahua, and the tactile practices of sowing or preparing ritual foods, which reinforce ecological knowledge through embodied experience. In this way, food-centred festivals function not only as ritual observances but also as cultural mechanisms through which environmental relationships are experienced, remembered, and transmitted across generations.

A detailed observation is mentioned below:

- ***Aakha Teej*/** ***Akati***: **Blessings in the bowl of Palash**^10^**leaves**

Akha Teej, locally known as Akati, marks the beginning of the agricultural year among the Gond and Baiga communities of the Maikal Hills. When asked about the rituals associated with agriculture, one respondent explained about this festival and said, “*Akha Teej* marks the beginning of our new year. It is a day when we honour the land, the gods residing in nature, and our ancestors.” During the celebration, each household prepares a small bowl made from palash leaves and fills it with paddy grains stored from the previous harvest. These bowls are then carried collectively to the shrine of the village guardian deity located at the boundary of the settlement. He said, “We walk together to *Thakur Dev*’s^11^ shrine at the village boundary, singing songs and offering our harvest.” *Akha Teej* is not just about sowing seeds in the soil; it is about sowing respect, gratitude, and reverence for the earth and its spirits.”

At the shrine, the village priest^12^ performs ritual prayers and blesses the paddy grains offered by community members. After the ritual, the grains are returned to each household and preserved until the onset of the monsoon, when they are sown in the fields as the first seeds of the new agricultural cycle. According to local belief, these ritually blessed grains ensure agricultural prosperity and protection for the crops during the growing season. Respondents emphasised that the ritual symbolises respect and gratitude toward the earth and the spiritual forces believed to sustain agricultural life. One elder explained that the festival represents not only the beginning of sowing but also a reaffirmation of the community’s relationship with the land and its natural elements.

Field observations indicate that the ritual symbolises more than the practical preparation of seeds. The use of palash-leaf bowls and the collective offering of paddy grains emphasise the community’s relationship with both cultivated land and surrounding forests. The ritual, therefore, functions as a symbolic act of gratitude toward the earth and the natural forces believed to sustain agricultural life. Such practices reflect what environmental anthropology describes as a relational understanding of nature, where humans, land, and spiritual forces are interconnected within a shared ecological system (Descola 1996; [21]). Through this ritual, *Akati* marks the transition into a new agricultural cycle while reaffirming the community’s moral responsibility to respect and protect the land that sustains their livelihood. In this sense, the ritual use of paddy during *Akati* reflects a broader worldview in which food, ritual practice, and environmental relationships are closely interconnected.

- ***Bidari***: **The sacred sowing of seeds**

The subsequent conversation with the elderly individuals helped us document the sacrificial rituals associated with such occasions. Bidari is a significant agricultural celebration held in the Hindu months of *Jethh* (May–June) or *Asharh* (June–July), just before the sowing season; it marks the ceremonial preparation of seeds for cultivation. During the ritual, community members gather with selected seeds, often millets and other local grains, which are collectively blessed before being sown in the fields. Songs performed during the festival invoke the earth, crops, and natural forces associated with agricultural fertility. These songs function as expressions of hope for a successful harvest and reaffirm the community’s dependence on the land.

In preparation, the women of the household clean and purify their homes and courtyards by smearing them with a paste of cow dung, a common ritual act of sanctification. A song related to this festival was recorded by the first authors on.

*Mahua phoolva jhare re*,

*Darra-darra gao jaage re.*

*Mandar baja*,* lor re lor*,

*Nache baiṅgā mana ghana jor.*

*Ai jangal māi*,* rākho raa*,

*Karma pāg re chānndni taa.*

*Rāti ujjar*,* āg re jhāl*,

Gaao sangwa—dil hoye khāl.

The song can be translated as-.

Mahua flowers fall softly,

The village stirs awake.

The drum beats—echo, echo,

The Baiga folk dance with all their heart.

O Mother Forest, keep us safe,

Guide our steps in the Karma dance.

Tonight is bright with firelight,

We sing together, our hearts open.

On the day of the festival, *Baiga* priests bring seeds of grains and vegetables such as *Kodo*, *Kutaki*, *Kanagni*, *Jowar*, *Bajra*, *Rahar*, and Maize in leaf-cups made from the *Mahlon* tree. These are displayed at *Mukaddam*’s house. “The *Dawar* (village priest) starts by worshipping Mother Earth. The seeds are mixed and arranged in a semi-circular shape called *Ras*, and the villagers place onions in each *Ras*. The *Guniya* (traditional healer) then performs *Hom-Dhoop*^13^ using *Sarai-lasha* (resin from the *Sarai* tree), and the group worships chickens of various colours, each representing different spiritual forces. Notably, all gods and goddesses except *Narayan Deo*^14^ are worshipped during this ritual. That night, the Baiga observe a fast.

Sacrificial offerings are a key part of the ritual. A black hen is offered to *Dharti-Mata* (Earth Mother), a spotted (*Kabari* in local dialect) hen to *Kuwari Mata*^15^, and a white hen to *Thakur Deo*. Additionally, coconut and milk are presented to *Khairmai*^16^. “Thakur Deo and *Khairmai* are worshipped outside the village at their shrines. *Ras* liquor is sprinkled during the rituals, while the *Guniya* recites mantras. The *Dawar* drinks the remaining liquor. Then we drop the fresh blood of the hens into the *Ras*.” Finally, the sacred grains are mixed and distributed; each Baiga family receives one leaf-cup of these seeds to sow in their fields. The community believes that planting these sanctified seeds ensures a bountiful harvest, keeps wild animals away, and protects the crops from diseases.

During field observations, it was noted that the songs sung during Bidari frequently refer to the earth as a nurturing entity and emphasise the responsibility of humans to respect and protect the land. In this context, the purpose of the song is not only celebratory but also pedagogical, reminding participants of their moral and ecological relationship with the environment. Such expressions reflect what environmental anthropology describes as a relational understanding of nature, where human survival is closely linked to ecological balance (Descola 1996; [21]). At the same time, the performance of songs during the ritual creates a shared sensory experience. The collective rhythm of singing and the repetition of agricultural themes reinforce ecological knowledge and seasonal awareness within the community. From the perspective of sensory anthropology, these practices illustrate how environmental knowledge is transmitted through embodied and auditory experiences that connect cultural memory with everyday subsistence activities [8, 20, 28]. Through this combination of ritual action and song, Bidari serves both as a symbolic invocation of agricultural fertility and as a cultural mechanism for transmitting ecological knowledge.

- ***Hareli*/** ***Hariyali***: **Forest offerings and household blessings**

Hareli, also known as Hariyali, is celebrated during the Hindu month of *Savan* (July–August) on the day of *Amavasya* (new moon). Among the Gond and Baiga communities of the Maikal Hills, the festival marks an important moment in the agricultural calendar and symbolises the community’s relationship with both cultivated land and surrounding forests. On this day, the head of each household goes into the forest to collect specific plants and materials, such as bamboo, *jhiri*, boughs of Bhilawa, roots of Jogi Lati, *hasia thapar*, and branches of Bhawarmal. These materials are brought back to the village and used in ritual practices associated with agricultural protection and household well-being.

The collected plants are taken to the fields, where the head of the household performs a ritual offering to *Dharti Mata* (Mother Earth). The branches are then fastened into the soil as symbolic markers of fertility and protection. Some of these sacred items are also placed on rooftops and in cowsheds to safeguard the household and livestock. The village *Dawar* (ritual priest) visits homes and fastens a ritual branch in each household, reinforcing the collective nature of the ceremony. The plants used in the ritual are locally understood to possess protective and symbolic properties. Bamboo represents strength and continuity, while Bhilawa branches are associated with warding off harmful influences. The roots and branches gathered from the forest reflect the community’s dependence on forest ecosystems for both subsistence and spiritual protection. The ritual placement of these plants in fields and houses symbolically extends the forest’s protective power into cultivated and domestic spaces.

Food preparation also forms an important part of the celebration. Households prepare a sweet dish known as *Babara*, which is shared within the family and among neighbours. The festival also includes the feeding of domestic animals, which are given Mahlon leaves sprinkled with salt. This practice symbolises care for livestock that play an essential role in agricultural life. The inclusion of animals in the ritual reflects the broader understanding that agricultural prosperity depends on harmonious relationships between humans, animals, land, and forest resources.

Field observations indicate that Hareli functions as both an agricultural and ecological ritual. By incorporating forest plants into household and farming spaces, the ceremony symbolically acknowledges the interconnectedness of forest ecosystems and agricultural livelihoods. Such practices reflect what environmental anthropology describes as a relational understanding of nature, in which humans, landscapes, and spiritual forces are embedded within a shared ecological system (Descola 1996; [21]). The festival, therefore, represents not only a celebration of agricultural prosperity but also a reaffirmation of the community’s ethical and ecological relationship with the natural environment.

Hareli is followed approximately two months later by the *Pola*^17^ festival, which further celebrates agricultural labour and expresses gratitude toward animals and natural forces that support farming activities. Together, these festivals form part of a seasonal ritual cycle through which Gond and Baiga communities maintain their relationship with the land and its ecological rhythms.

- ***Bhojali***: **Sprouting gratitude and growth**

Bhojali is a monsoon festival observed among the Gond and Baiga communities in the study region. The ritual centres on the symbolic germination of grains and is closely associated with fertility, agricultural renewal, and ecological balance. Preparations begin seven to nine days before the full moon of the month of *Savan*. During this period, women, particularly young girls, collect clean soil from a sacred place, often near a river or pond, and place it in small earthen pots. Seeds of wheat or barley are then sown in the soil and watered daily until they sprout.

**Fig. 4:**
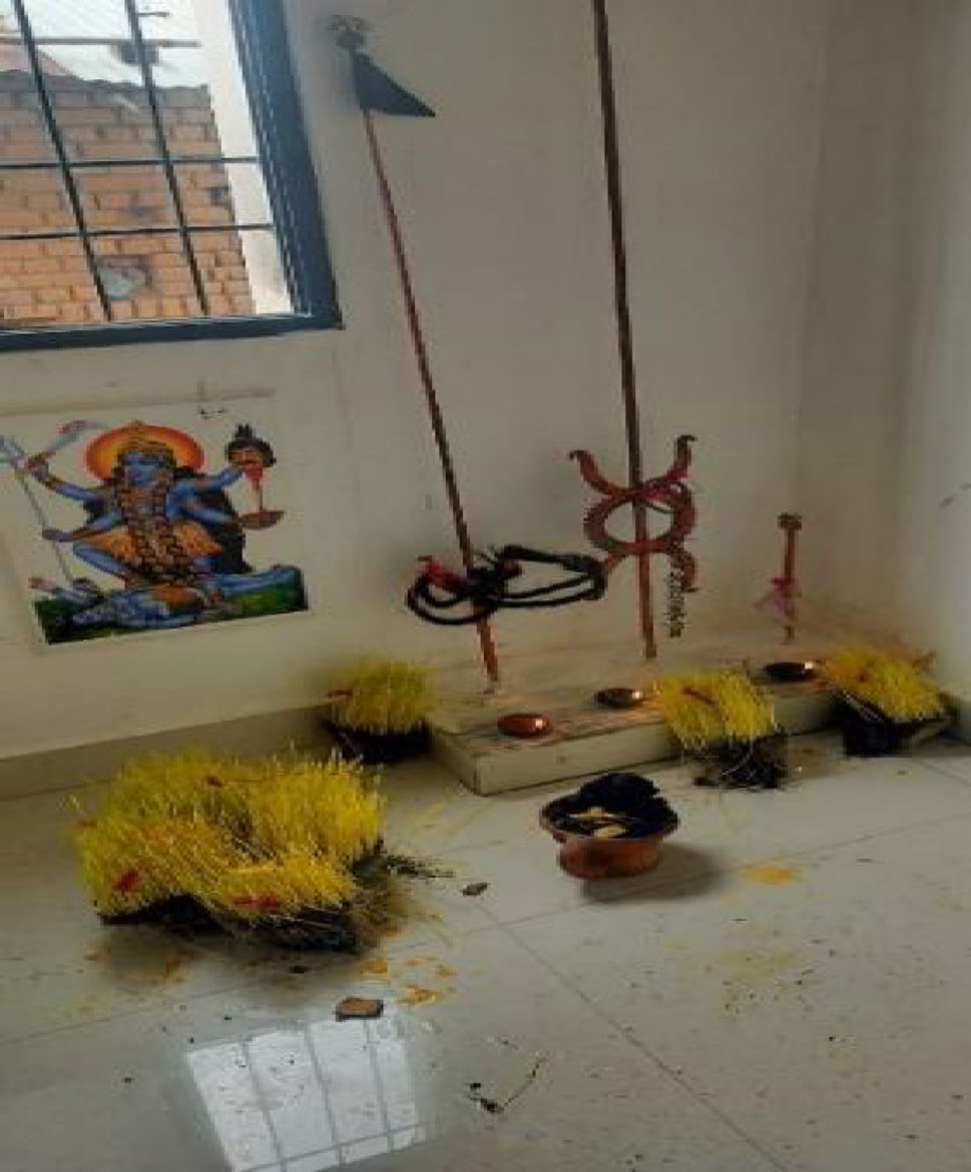
Celebrating Bhojali in a Gond house

Respondents explained that wheat and barley are considered ancient and auspicious grains within local traditions. Their rapid germination and vibrant green shoots are interpreted as signs of fertility, agricultural prosperity, and the earth’s regenerative capacity. During the days leading up to the festival, young girls gather around the pots, water the sprouting grains (Fig. 4), and sing songs dedicated to natural forces such as rain, soil, and vegetation. These songs serve both as expressions of gratitude toward nature and as cultural practices through which younger generations learn about seasonal cycles and agricultural values.

On the final day, which coincides with *Savan Purnima*, the sprouted Bhojali plants are carried in a small procession to a nearby river or pond. Community members sing and dance as the plants are immersed in flowing water. The immersion symbolically returns the life force of the germinating grains to nature, expressing gratitude for fertility and continued rainfall.

Our observations suggest that Bhojali functions as a ritual metaphor for agricultural regeneration. The act of nurturing sprouting grains in earthen pots reflects the broader agricultural process of seed germination and crop growth. At the same time, collective singing, plant watering, and ceremonial immersion create sensory and embodied experiences through which ecological knowledge is transmitted. From the perspective of environmental anthropology, such practices illustrate a relational understanding of nature in which human livelihoods, agricultural cycles, and natural forces are closely interconnected (Descola 1996; [21]). The sensory engagement involved in nurturing the sprouting plants and participating in the ritual also reflects the role of embodied practices in transmitting environmental knowledge across generations [8, 20, 28].

Through this ritual, Bhojali becomes more than a seasonal celebration; it represents a cultural expression of ecological awareness, agricultural hope, and community participation. The sprouting grain, therefore, serves as a powerful symbol of growth, renewal, and gratitude within the indigenous worldview of the Maikal Hills.

- ***Chherata***: **Children’s festival and agricultural myth**

**Fig. 5:**
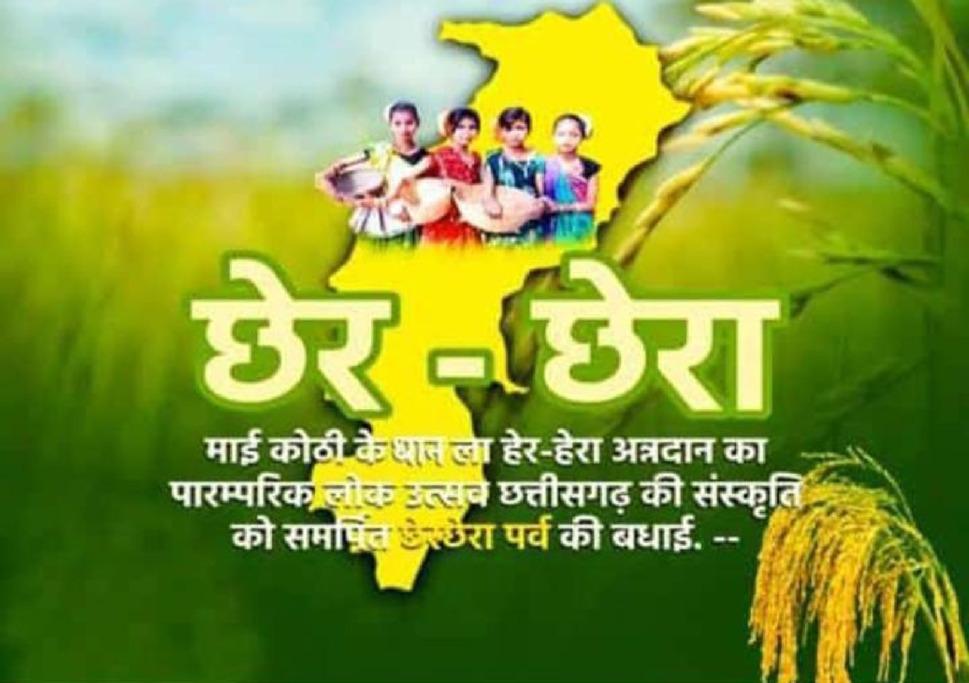
A poster for celebrating Chherata

Chherata is celebrated during the winter season on the full moon day (*Purnima*) of the Hindu month of *Poos* (December–January). Among the Gond and Baiga communities, the festival is primarily associated with children and communal food sharing. On this occasion, children carry sticks, dress in simple symbolic attire, and move from house to house asking for *Chherata*,^18^ a term locally understood to mean alms or charitable offerings (Fig. 5). As they walk through the village, they sing a chant, *“Chher Chhitta Chherata*,* Mai Kothi ke Dhan Herta*,* Chher Chhitta Chherata.”* The chant invokes the earth and asks for grains, symbolically expressing the community’s dependence on agricultural abundance.

In response to the children’s requests, households offer rice, pulses, and *kudai* (parched grains). After collecting these food items, the children gather near a water body where they bathe and cook the collected grains together. Before eating, a portion of the food is offered to local deities, reflecting the cultural norm that food must first be acknowledged as a gift from the earth and spiritual forces. A distinctive element of the ritual involves selecting a boy to represent a crow. He eats first, after which a girl throws a burning stick toward him, prompting him to run away. This action signals the beginning of the communal meal and symbolically marks the transition from ritual offering to collective consumption.

Respondents also associate the festival with an agricultural myth related to the origins of *Bewar* (shifting or slash-and-burn cultivation). According to local oral tradition, an ancestral figure known as Nanga Baiga once witnessed a destructive fire that burned the vegetation of Jambu Dweep. Distressed by the loss of crops and vegetation, he repeatedly struck the scorched earth while crying out, *“Chher Chher Chherata.”* In response to his suffering, the earth appeared in the form of a woman and instructed him to sow seeds in the holes created by his strikes. From these holes, crops began to grow again, marking the beginning of *Bewar* cultivation.

Field observations suggest that this myth functions as a cultural explanation for agricultural regeneration following forest fires, a process closely associated with shifting cultivation practices. The chant and ritual actions performed by children symbolically recreate this narrative, linking agricultural fertility with the earth’s regenerative power. From the perspective of environmental anthropology, such myths illustrate a relational understanding of the environment in which humans, land, and spiritual forces are interconnected (Descola 1996; [21]). At the same time, the active participation of children in collecting, cooking, and sharing food creates a sensory and experiential context through which agricultural knowledge and cultural values are transmitted across generations.

Through these practices, Chherata functions both as a playful community celebration and as a symbolic reminder of the origins of agricultural life, emphasising the importance of cooperation, gratitude toward the earth, and the cyclical renewal of crops within the indigenous worldview.

- ***Nawakhani***: **The first taste of the food**

Nawakhani is a harvest festival that marks the ceremonial tasting of newly harvested crops among the Gond and Baiga communities of the Maikal Hills. Unlike many festivals fixed to a specific date, Nawakhani is celebrated at a convenient time during the month of *Bhado* (July–August), depending on the readiness of the crops.^19^ The ritual centres on offering the first portion of the harvest to ancestral spirits before it is consumed by the household.

Preparations for the ritual begin a day before the celebration. The head of the household visits the field and collects cucumber flowers in the names of local deities. These flowers are placed on small wooden pegs as symbolic offerings, acknowledging the spiritual forces believed to protect crops and agricultural fertility. On the day of the festival, the house is thoroughly cleaned, and the floor is smeared with cow dung, a practice widely understood in rural communities as a form of ritual purification. Newly harvested grains are then cooked together with rice, pulses, and vegetables to prepare a ceremonial meal.

Respondents explained that consuming the new crop without performing the ritual offering is considered inappropriate and potentially dangerous. Such beliefs reinforce the moral obligation to acknowledge the sources of agricultural sustenance before benefiting from them. The ritual, therefore, expresses gratitude toward the land and ancestral spirits while symbolically ensuring protection and prosperity for the household.

Songs performed during the ceremony further emphasise these relationships. One such song is sung while the Baiga perform *hom-dhoop* in the name of their ancestors:

*Le aja dadi saha ho*,

*Kata kuti na karo.*

Bag bhalu door kar.

*Tumnak pahunche hoomal.*

The song can be translated as-.

Come here, grandmother,

take this and do not cut anything.

Keep the bear away so that.

misfortune does not come to us.

The song invokes ancestral protection and expresses hopes for safety from wild animals and other threats that could damage crops or harm the community. In this context, the performance of the song functions both as a ritual invocation and as a cultural expression of the community’s dependence on natural forces and ancestral spirits.

Our observation indicates that Nawakhani symbolically reinforces the principle that food must first be offered to ancestral and spiritual forces before being consumed by humans. Such practices reflect what environmental anthropology describes as a relational understanding of nature, where human survival, agricultural cycles, and spiritual beliefs are closely interconnected (Descola 1996; [21]). At the same time, collective singing, ritual cooking, and food offerings create sensory experiences that embed ecological knowledge and cultural memory in everyday practices [8, 20, 28].

Through these rituals, Nawakhani becomes more than a harvest celebration; it represents a cultural expression of gratitude, protection, and reciprocity between humans, their ancestors, and the natural environment that sustains agricultural life.

- ***Javanra***: **The ritualisation of the agrarian life**

Javanra is an important ritual festival associated with Shakti worship and the agricultural cycle in the studied region. The festival is usually observed during the month of *Kunwar* (March–April) in the *Shukla Paksha* (bright fortnight) of the Hindu calendar. Local respondents explained that this period coincides with seasonal transitions in the agricultural calendar, when preparations for cultivation begin, and communities seek blessings for fertility, crop growth, and collective well-being. The festival, therefore, represents a moment when spiritual practices and agricultural life intersect.

At the centre of the ritual is the installation of the *Jyoti Kalash*, a sacred vessel containing a continuously burning lamp (*Akhand Jyoti*). The ceremony begins with the ritual worship of soil, which is regarded as sacred because it sustains agricultural life. Soil is collected from a selected site after prayers and placed in a newly woven bamboo basket. Barley and wheat seeds are then sown in this soil, symbolising the regenerative cycle of agriculture and the hope for a successful harvest. The sprouting of these seeds during the nine days of the ritual is interpreted as a sign of fertility and renewal.

During this period, strict ritual observances are maintained. Local priests (*pandas*) follow dietary restrictions, refrain from consuming meat, and dedicate themselves to daily prayers. Women participate by singing *Jas Devigeet*, devotional songs praising the goddess and seeking protection for the community and its crops. One such song expresses devotion and humility before the divine:

*Jiyat jiyara tor dham mata*,

*Khanihari dharela aahun.*

Marat piyara tor dham ma.

*Dev mile la aahun.*

The song can be translated as-.

Mother, while I am alive, I come to your sacred place with devotion.

I come as a humble labourer who gathers food through work.

Even in death, beloved Mother,

I will return to your holy place, where I will meet the divine.

The songs sung during the ritual emphasise devotion, humility, and dependence on divine protection. In this context, the performance of devotional songs functions as both a spiritual invocation and a cultural expression of gratitude for agricultural sustenance.

The final day of the festival involves the immersion of the *Javanra*, consisting of sprouted grains and sacred soil, in a nearby river or stream. This act symbolises the return of sacred fertility to nature. During the immersion procession, participants carry the *Jyoti Kalash* on their heads while singing and dancing. Along with this, thousands of worshippers of *Shakti* wear *Bana* and *Sanga*, made of iron (a long, pointed spear worn by inserting it across the body), on their cheeks and bodies, and walk in the procession smiling, singing, and dancing. The procession culminates in a communal gathering where ritual food prepared from the harvest is shared among participants. A communal feast follows, where all food prepared from the harvest is shared. A respondent reflected, “This food is not ordinary. It is *prasadi*^20^. Everyone eats together, and it reminds us that what we grow is also what we offer to the gods. There is no separation.” For the villagers, *Javanra* is more than a religious festival. It is an annual moment of renewal that connects land, labour, and the divine. It marks a time when spiritual life and agricultural life converge. As one farmer explained, “These rituals tell us the time, when to begin and give thanks to the natural forces so farming is not just work for us; it is part of our dharma.”

Field observations suggest that *Javanra* functions as a ritual expression of agricultural renewal and collective gratitude toward natural and spiritual forces. The symbolic sowing of seeds, the nurturing of sprouting grains, and the final immersion in water reflect a cyclical understanding of life, fertility, and regeneration. From the perspective of environmental anthropology, such rituals illustrate a relational worldview in which humans, land, and spiritual forces are closely interconnected (Descola 1996; [21]). The collective singing, ritual performances, and communal meals also create sensory and embodied experiences that reinforce cultural memory and environmental awareness [8, 20, 28].

For community members, *Javanra* therefore represents more than a religious observance. It marks a moment of renewal in which agricultural labour, spiritual devotion, and social solidarity converge. Through these practices, the festival reaffirms the cultural understanding that farming is not merely an economic activity but an ethical and spiritual relationship with the land.

## Discussion

### Ecological relationships in food-centred festivals

After examining the information related to the food-centred festivals of the Gond and Baiga people of central India, it becomes evident that indigenous societies live through and with their environments, reaffirming the ecological intimacy highlighted in environmental anthropology (Descola 1996; [21]). These festivals are not merely calendrical events but are deeply rooted in agricultural and ecological cycles, marking seasonal transitions such as sowing, crop growth, harvest, and post-harvest renewal (Table 3). As Elwin [12] observed, Adivasi ritual calendars themselves function as ecological maps synchronised with monsoon rhythms, crop maturation, and the flowering patterns of forest species. Through such celebrations, communities articulate their dwelling within the land rather than acting upon it as an external resource.

**Table 3:** Ecological cycles and associated food-centred festivals among Gond and Baiga communities

Festival	Agricultural stage	Key ritual practice	Ecological meaning
*Aakha Teej* (*Akati*)	Beginning of agricultural year	Offering paddy seeds to village deity	Invocation of agricultural fertility
*Bidari*	Pre-sowing season	Ritual blessing and distribution of seeds	Protection of crops and ecological balance
*Hareli*	Monsoon agricultural cycle	Worship of farming tools and forest plants	Recognition of earth and agricultural labour
*Nawakhani*	Harvest period	Offering first grains to ancestors	Gratitude for agricultural abundance
*Javanra*	Seasonal agricultural transition	Germination of ritual seeds	Symbolic regeneration and fertility

Several festivals documented during fieldwork illustrate this ecological relationship. In ceremonies such as *Bidari* and *Nawakhani*, newly harvested crops or ritual seeds are first offered to deities or ancestors before being consumed by the community. This practice symbolises respect for natural forces and acknowledges the dependence of human life on the land. Similarly, festivals such as *Hareli* and *Javanra* involve the ritual use of plants, seeds, and agricultural tools, emphasising the interconnectedness of farming practices, forest ecosystems, and spiritual beliefs. These events function not only as religious observances but also as cultural mechanisms through which ecological knowledge is reinforced and transmitted within the community.

### Symbolism of ritual foods and sensory ecologies

In festivals such as *Hareli*, *Bidari*, *Bhojali*, and *Javanra*, the preparation and offering of foods including millets, rice, tubers, mahua, and forest fruits serve as expressions of gratitude to the land and ancestors. These foods are not selected merely for their availability; they embody relational ethics that structure human–nature ties. Millets grown in shifting fields, mahua flowers gathered in forest groves, and wild tubers dug from ancestral grounds carry what Ingold [21] describes as traces of environmental co-presence, material evidence of a life lived in reciprocity with the landscape. Food therefore becomes a medium through which ecological continuity is honoured and renewed. The symbolic resonance of these ingredients lies not only in their nutritional value but also in their capacity to evoke the land, its cycles, spirits, and ancestral histories [38].

The specific foods used in these festivals also reflect the ecological conditions of the Maikal Hills region. Millets such as kodo and kutki are drought-resistant crops well suited to the upland agricultural systems of central India. Their ritual use highlights their historical importance in indigenous food systems and demonstrates the resilience of local cropping traditions (Table 4). Forest-based foods such as mahua flowers, wild tubers, and seasonal fruits also occupy an important place in ceremonial practices and communal meals. Mahua, for instance, is used both as food and as a fermented beverage during ritual gatherings, reinforcing its economic and cultural significance in local life.

**Table 4:** Symbolic and ecological meanings of ritual foods used in festivals

Ritual food	Source	Symbolic meaning	Ecological significance
Millets (kodo, kutki)	Cultivated upland crops	Agricultural resilience and subsistence continuity	Drought-resistant crops suited to local ecology
Mahua flowers	Forest resource	Sacred food and ceremonial beverage	Reflects forest dependence and seasonal gathering
Wild tubers	Forest soils	Ancestral food and subsistence resource	Indicates knowledge of forest ecosystems
Rice/grains	Cultivated fields	Harvest gratitude and ritual purity	Represents agricultural abundance
Forest fruits	Wild forests	Seasonal nourishment and ritual offering	Symbolises biodiversity of local forests

Sensory anthropology further illuminates how these festivals embed environmental knowledge and cultural memory through embodied experiences. The aroma of roasted kodo millet in *Hareli*, the sticky sweetness of fermenting mahua in *Bidari*, the tactile act of clearing the soil, or the rhythmic sound of pounding rice are more than sensory details; they function as mnemonic practices that encode ecological familiarity [8, 13, 20]. These sensory cues are transmitted across generations, enabling younger members of the community to internalise the feel, smell, and taste of their landscape. Sensory practices also guide subsistence decisions, such as determining soil moisture through touch during sowing, recognising tuber ripeness through scent, or interpreting the sound of rivers during fishing activities.

Our ethnographic findings from villages of the Maikal Hills illustrate how these ideas materialise in lived practice. During the Gond festival of *Nawakhani*, the first produce of the season, often kodo-kutki dishes, is offered to deities before being shared communally. The sensory process of preparing these foods mirrors environmental rhythms: the dampness of monsoon air, the smell of new shoots, and the texture of freshly harvested grains. Similarly, the Baiga festival of *Bidari* foregrounds forest dependence through dishes made from tubers, fruits, and leaves gathered from uncultivated landscapes. These foods carry the taste of the forest, reinforcing the Baiga perception of the forest as kin and protector [30, 37].

Ritual songs and dances performed during these festivals further strengthen this sensory–environmental connection. Songs describing the smell of first rains, the sound of birds announcing crop readiness, or the taste of mahua nectar anchor cultural identity in embodied ecological memory. Such expressions reveal what Pink [28] describes as embodied emplacement, where the body becomes the site through which environmental belonging is experienced. Similarly, the pounding of grain, the woody fragrance of roasting mahua, and the collective hum of song during meal preparation enact what Sutton [38] describes as the mnemonic potency of the senses, turning kitchens and courtyards into archives of cultural memory.

### Comparative perspectives on indigenous food festivals

The food-centred festivals documented in the Maikal Hills can be better understood when viewed within a broader context of indigenous food traditions observed in different regions of India and beyond. Several communities across South Asia celebrate festivals associated with the first harvest, ritual offerings of crops, and communal feasting that express gratitude to nature and ancestral spirits. For instance, festivals such as *Nuakhai* in Odisha, *Nabanna* in West Bengal, and *Wangala* among the Garo community of Northeast India similarly mark agricultural cycles and the consumption of newly harvested grains after ritual offerings to deities or ancestors (Table 5). Comparable practices are also observed in other indigenous cultures worldwide, such as *Pachamama* offerings among Quechua communities in the Andes, where crops like maize and potatoes are ceremonially offered to Mother Earth before agricultural activities begin.

**Table 5:** A comparative perspective on festivals associated with the first harvest across India and other parts of the world

Region/Community	Festival	Ritual food	Cultural meaning
Central India (Gond–Baiga)	Nawakhani	Millets/new grains	First harvest offering to ancestors
Odisha	Nuakhai	Rice	Celebration of new harvest
West Bengal	Nabanna	Rice dishes	Agricultural prosperity
Northeast India (Garo)	Wangala	Rice/grains	Thanksgiving to agricultural deities
Andes (Quechua)	Pachamama rituals	Maize, potatoes	Reciprocity with Mother Earth

However, the festivals documented in the Maikal Hills region display several distinctive characteristics shaped by the ecological conditions and subsistence patterns of the Gond and Baiga communities. Unlike many agrarian societies where rice occupies a central ritual position, several festivals in this region emphasise millets such as kodo and kutki, which are well-suited to the upland ecological conditions of central India. In addition, forest-based foods such as mahua flowers, wild tubers, and gathered fruits play an important role in ritual meals and offerings. These elements reflect the continuing dependence of these communities on forest ecosystems and highlight a subsistence system that integrates agriculture with forest gathering.

Previous studies on indigenous communities in central India and neighbouring regions have documented several agricultural and harvest-related festivals associated with ritual food practices [3, 23, 26]. To situate the findings of the present study within the broader ethnographic literature, a comparison was conducted between festivals documented during the fieldwork in the Maikal Hills and those reported in earlier research from other regions of India. This comparison highlights both the shared cultural patterns associated with ritual food practices and the distinctive features of Gond and Baiga food festivals documented in this study.

**Table 6:** Similarities and differences between the festivals associated with the first harvest

Region/Community	Festival	Main ritual foods	Main ritual practices	Commonalities with maikal hills festivals	Key differences	Cultural interpretation
Central India (Gond & Baiga – Maikal Hills)	*Nawakhani*, *Bidari*, *Hareli*, *Javanra*	Millets (kodo, kutki), maize, mahua, forest tubers, newly harvested grains	First harvest offerings, seed blessing, ritual sowing, communal feasts	Strong ritual connection between agriculture, food, and spiritual beliefs	Greater emphasis on millets and forest foods rather than rice	Reflects ecological adaptation to upland forest landscapes and mixed subsistence strategies
Odisha (Western Odisha communities)	*Nuakhai*	Newly harvested rice	First consumption of new rice after offering to deities and ancestors	Celebration of first harvest and gratitude to land and ancestors	Dominance of rice as staple crop	Agricultural rituals closely tied to irrigated rice cultivation systems
West Bengal (agrarian communities)	*Nabanna*	Fresh rice, rice-based dishes	Ritual preparation of newly harvested rice and communal feasts	Harvest celebration and collective sharing of food	Limited role of forest foods	Symbolises agrarian prosperity and community solidarity
Northeast India (Garo community)	*Wangala*	Rice, millet, local grains	Ritual dances and thanksgiving to agricultural deities	Expression of gratitude for harvest and agricultural cycles	Greater emphasis on musical rituals and large communal dances	Agricultural festivals reinforce community identity and ecological balance
Andes (Quechua communities)	*Pachamama* offerings	Maize, potatoes, grains	Ritual offerings to Mother Earth before agricultural activities	Reciprocity between humans and nature through food offerings	Different crops reflecting high-altitude agriculture	Demonstrates universal indigenous cosmologies linking food, land, and spirituality

The comparative perspective also highlights both commonalities and differences in the cultural meanings associated with ritual food practices (Table 6). Across regions, harvest festivals express a shared principle of reciprocity between humans and the natural environment, in which food is offered to divine or ancestral forces before being consumed by the community. At the same time, the specific crops and ritual forms vary according to ecological conditions and local cultural traditions. While rice symbolises agricultural abundance in eastern India, the prominence of millets and forest foods in the Maikal Hills reflects a landscape where mixed farming and forest-based livelihoods remain central to community life. These differences reflect the forested upland ecology of central India and the mixed subsistence strategies that combine agriculture with forest gathering.

A simple frequency comparison of ritual food types mentioned in the documented festivals further highlights the distinctive characteristics of the Maikal Hills traditions. Among the seven festivals recorded during fieldwork, millets and local grains appear in approximately 57% of the rituals, while forest-derived foods such as mahua, tubers, and gathered fruits appear in about 29% of the cases, and newly harvested grains account for around 14% of ritual offerings.^21^ In contrast, previous studies of harvest festivals in eastern India report a predominance of rice-based ritual foods in more than 70% of documented ceremonies [3, 23]. This comparison indicates that while many indigenous festivals share the broader theme of first-harvest rituals, the Gond and Baiga celebrations place greater emphasis on millets and forest resources, reflecting the ecological conditions and subsistence patterns of the Maikal Hills region.

As this study is based on ethnographic fieldwork conducted in selected Gond and Baiga settlements of the Maikal Hills region, and the findings therefore reflect the cultural practices documented within this specific geographical context. By emphasising indigenous crops such as millets and forest produce like mahua, these festivals demonstrate culturally embedded practices that sustain traditional food systems and reinforce collective relationships with land, forests, and ancestral traditions. While the research provides detailed insights into several food-centred festivals, it does not aim to represent all indigenous communities of central India or the full diversity of their ritual traditions. In addition, the statistical comparison presented in the study is based on a limited number of documented festivals and is intended primarily as an illustrative tool to highlight patterns in ritual food use rather than as a comprehensive quantitative analysis. Future research could expand the geographical scope of fieldwork and incorporate larger datasets to explore regional variations in indigenous food festivals and ritual food systems across central India.

### Food sovereignty and revitalisation of indigenous food heritage

These observations have important implications for discussions of indigenous food sovereignty. The continued ritual use of traditional crops such as millets and forest-derived foods contributes to the preservation of local food systems that are adapted to the ecological conditions of the Maikal Hills (Table 7). By prioritising these ingredients during ceremonial events, communities maintain knowledge of traditional cultivation practices, local seed varieties, and seasonal gathering strategies. Such practices support agricultural biodiversity and strengthen food systems that are less dependent on external agricultural inputs.

The festivals also function as cultural spaces through which ecological knowledge and food traditions are transmitted to younger generations. Participation in ritual activities such as seed preparation, food preparation, and communal feasting allows younger members of the community to learn about the cultural significance of indigenous crops and forest foods. In this way, ritual practices contribute to revitalising traditional food heritage at a time when market-based diets and agricultural modernisation are transforming rural food systems.

**Table 7:** Role of food-centred festivals in sustaining indigenous food sovereignty

Cultural practice	Knowledge transmitted	Contribution to food sovereignty
Ritual preparation of millets	Traditional cultivation methods	Preservation of indigenous crops
Forest food gathering	Ecological knowledge of forests	Protection of biodiversity
Communal cooking during festivals	Cultural transmission of recipes	Maintenance of traditional diets
Ritual seed blessing	Seed preservation practices	Strengthening local seed systems
Intergenerational participation	Cultural education	Revitalisation of food heritage

Revitalising this heritage, therefore, requires recognising the cultural value of indigenous food traditions and supporting the communities that sustain them. Documentation of ritual food practices, promotion of traditional crops such as millets, and protection of forest-based livelihoods can contribute to safeguarding these traditions. Policies that encourage local seed systems, community-based conservation, and the recognition of indigenous ecological knowledge may further strengthen these culturally embedded food systems. The food-centred festivals of the Maikal Hills thus represent not only expressions of cultural identity but also locally grounded approaches to ecological sustainability and indigenous food sovereignty.

## Conclusion

Overall, the food-related festivals of the Maikal Hills are far more than agricultural customs; they are enactments of a sacred ecology in which land, labour, and lineage are woven into ritual life. In these ceremonies, the community is reaffirming its connection to the earth, asking for protection from the forces of nature, and maintaining cultural history. These rituals, whether they are the mythical screams of *Chherata* or the ceremonial planting of *Bidari*, demonstrate a worldview that suggests food is not just grown, it is given by the earth, bartered with spirits, and shared with ancestors. In this context, foodways emerge as both *survival strategies* and *ritual acts*.

In all senses, the food-centred festivals of the Gond and Baiga communities in central India’s Maikal Hills, as explored in this study, are profound expressions of cultural resilience, ecological harmony, and social cohesion. Far beyond mere agricultural customs or celebratory feasts, festivals such as *Aakha Teej*, *Bidari*, *Hareli*, *Bhojali*, *Chherata*, *Nawakhani*, and *Javanra* weave together land, labour, lineage, and spirituality into a sacred ecology. Through a mixed-methodological approach combining ethnographic fieldwork and theoretical frameworks, this research illuminates how these festivals embody a holistic worldview in whifch food serves as both sustenance and a powerful symbol of cultural and ecological interconnectedness. By honouring agricultural cycles, local biodiversity, and ancestral traditions through offerings of millets, mahua, and forest produce, these communities reaffirm their symbiotic relationship with nature while resisting the homogenising forces of modernity, climate change, and land alienation.

Furthermore, this research significantly enriches anthropological scholarship by providing a nuanced exploration of how food practices encode cultural, spiritual, and ecological meanings, aligning with Lévi-Strauss’s [25] *culinary triangle* and Sarah Pink’s (2015) concept of *embodied emplacement*. It illustrates how food in festivals like *Aakha Teej* and *Bidari* serves as a cultural code articulating tribal relationships with nature and society, contributing to the anthropology of food by highlighting the symbolic and political roles of indigenous foodways [1, 11]. By prioritising local biodiversity and sustainable practices, the Gond and Baiga assert food sovereignty [34], offering a model for understanding indigenous resistance to environmental degradation and cultural assimilation. The ethnographic documentation of rituals, oral traditions, and material culture (e.g., clay pots, sacred groves) also serves as a valuable archive for cultural preservation, aligning with global efforts to safeguard intangible cultural heritage. Additionally, we can say the study’s further emphasis on communitas [39] and gendered roles in food preparation [9] will contribute to understanding social cohesion and women’s cultural authority in indigenous contexts, while its sensory and material culture analyses ([38]; Miller, 2005 [27]) have highlighted how festivals embed collective memory and identity.

In an era marked by environmental crises and a deepening alienation from the natural world, the festivals of the Gond and Baiga communities stand as powerful reminders of a different way of being, one rooted in reciprocity, reverence, and resilience. These celebrations are not merely cultural expressions; they are acts of gratitude, resistance, and identity that weave together food, ritual, and community into a living fabric of ecological harmony. By engaging with these festivals through symbolic, sensory, material, and political lenses, this study uncovers their profound role in sustaining both cultural continuity and ecological consciousness. As we face urgent global challenges, the wisdom embedded in these indigenous practices invites us to reimagine sustainability, not as a technical fix, but as a deeply relational ethics.

## Data Availability

No datasets were generated or analysed during the current study.

## Footnotes

1 Central India is frequently used to refer to the states of Madhya Pradesh and Chhattisgarh, situated at the centre of the country.

2 For the purposes of this article, the term ‘Tribal’ is used as a general descriptor for the studied Indigenous groups. The term is employed solely for clarity and consistency and is not intended to obscure the distinct identities, cultural specificities, or preferred self-designations of individual communities.

3 ‘Foodways’ refers to the cultural, social, and historical practices, beliefs, and meanings associated with the production, preparation, and consumption of food within a community.

4 This fieldwork was conducted by Akash Kumar Srivastava as part of his doctoral research.

5 Genetic research suggests that the Gond people, and by extension related groups like the Baiga, retain deep ancestral lineages and can be considered modern representatives of some of India’s earliest human populations.

6 The Maikal Hills stretch across the border regions of eastern Madhya Pradesh and northern Chhattisgarh, covering portions of several districts of both states. It is known for the Amarkantak, which is recognised as a key highland tract from which the Narmada River originates.

7 Snowball sampling is a technique in which participants refer other potential participants, useful for studying hidden or hard-to-access populations.

8 Ingold’s notion of ‘dwelling’ describes how humans are continuously immersed in and responsive to their surroundings, highlighting life as a process of active engagement and interaction with the environment rather than mere physical occupation of space.

9 Adivasi refers to India’s indigenous communities, meaning ‘original inhabitants,’ who have distinct cultures, languages, and traditional ways of life.

10 Scientifically known as *Butea monosperma*, Palash is a deciduous species native to the Indian subcontinent, noted for its vivid orange-red blossoms and its longstanding uses in traditional medicine, dye extraction, and ritual contexts.

11 A village-protecting deity venerated among several Adivasi communities, commonly understood as an ancestral or territorial guardian spirit invoked through collective rituals and offerings.

12 In many central Indian tribal communities, ritual duties during festivals are performed by hereditary village priests, commonly known as *Pahan*, *Dawar*, *Gaita*, or *Bhopa*, who act as intermediaries between the community, its deities, and ancestral spirits. In our case, the Baiga work as priests during these festivals.

13 This is the fragrant smoke from burning traditional herbal mixtures in tribal homes during festivals, used to purify the space and honor deities and ancestors.

14 He is a protective deity venerated across several central Indian tribal groups (including the Gond, Baiga, Korku, and Bharia), who is propitiated through distinctive rites, especially the sacrificial killing of a young pig at the household threshold, performed to secure protection from illness, misfortune, and harmful animals.

15 This is a virgin goddess in central Indian tribal life, worshipped for purity, protection, and the well-being of families and young girls.

16 This is a protective village deity in central Indian tribal traditions, often associated with forests and boundary guardianship, to whom offerings are made for safety, health, and communal well-being.

17
*Pola* is a central Indian tribal harvest-season festival in which working bullocks are washed, decorated, fed special offerings, and ritually honored in thanks for their role in agriculture.

18 This Baiga origin tale links the cry “*Chherata*” to the beginnings of *Bewar* cultivation, portraying how human distress, divine compassion, and the earth’s guidance shaped their traditional slash-and-burn farming.

19 Cow dung is used not only for its disinfecting and insect-repelling qualities but also for traditional reasons, as Indian society considers it purifying and believes it maintains ritual cleanliness within the home.

20 Sacred offering.

21 The percentages presented in this comparison are based on a simple frequency distribution of the seven festivals documented during fieldwork. Each festival was classified according to the dominant ritual food type involved in the celebration, and the proportion of festivals in each category was calculated relative to the total number of events recorded in the study.
